# Monitoring Low Molecular Weight Heparins at Therapeutic Levels: Dose-Responses of, and Correlations and Differences between aPTT, Anti-Factor Xa and Thrombin Generation Assays

**DOI:** 10.1371/journal.pone.0116835

**Published:** 2015-01-27

**Authors:** Owain Thomas, Emanuel Lybeck, Karin Strandberg, Nahreen Tynngård, Ulf Schött

**Affiliations:** 1 Medical Faculty, University of Lund, Lund, Sweden; 2 Department of Paediatric Anaesthesia and Intensive Care, SUS Lund University Hospital, Lund, Sweden; 3 Coagulation Laboratory, Dept. of Clinical Chemistry, Division of Laboratory Medicine, Skåne University Hospital, Malmö, Sweden; 4 Department of Clinical Immunology and Transfusion Medicine, Department of Clinical and Experimental Medicine, Linköping University, Linköping, Sweden; 5 Department of Clinical Chemistry, Department of Clinical Experimental Medicine, Linköping University, Linköping, Sweden; 6 Department of Anaesthesia and Intensive Care, SUS Lund University Hospital, Lund, Sweden; National Cancer Center, JAPAN

## Abstract

**Background:**

Low molecular weight heparins (LMWH’s) are used to prevent and treat thrombosis. Tests for monitoring LMWH’s include anti-factor Xa (anti-FXa), activated partial thromboplastin time (aPTT) and thrombin generation. Anti-FXa is the current gold standard despite LMWH’s varying affinities for FXa and thrombin.

**Aim:**

To examine the effects of two different LMWH’s on the results of 4 different aPTT-tests, anti-FXa activity and thrombin generation and to assess the tests’ concordance.

**Method:**

Enoxaparin and tinzaparin were added ex-vivo in concentrations of 0.0, 0.5, 1.0 and 1.5 anti-FXa international units (IU)/mL, to blood from 10 volunteers. aPTT was measured using two whole blood methods (Free oscillation rheometry (FOR) and Hemochron Jr (HCJ)) and an optical plasma method using two different reagents (ActinFSL and PTT-Automat). Anti-FXa activity was quantified using a chromogenic assay. Thrombin generation (Endogenous Thrombin Potential, ETP) was measured on a Ceveron Alpha instrument using the TGA RB and more tissue-factor rich TGA RC reagents.

**Results:**

Methods’ mean aPTT at 1.0 IU/mL LMWH varied between 54s (SD 11) and 69s (SD 14) for enoxaparin and between 101s (SD 21) and 140s (SD 28) for tinzaparin. ActinFSL gave significantly shorter aPTT results. aPTT and anti-FXa generally correlated well. ETP as measured with the TGA RC reagent but not the TGA RB reagent showed an inverse exponential relationship to the concentration of LMWH. The HCJ-aPTT results had the weakest correlation to anti-FXa and thrombin generation (R_s_0.62–0.87), whereas the other aPTT methods had similar correlation coefficients (R_s_0.80–0.92).

**Conclusions:**

aPTT displays a linear dose-respone to LMWH. There is variation between aPTT assays. Tinzaparin increases aPTT and decreases thrombin generation more than enoxaparin at any given level of anti-FXa activity, casting doubt on anti-FXa’s present gold standard status. Thrombin generation with tissue factor-rich activator is a promising method for monitoring LMWH’s.

## Introduction

Low molecular weight heparins (LMWH’s) are among the most-commonly used anticoagulants in modern healthcare, usually given in fixed doses once or twice daily. Although monitoring is not routine, it is recommended when accurate dosing is especially important, such as in the context of renal impairment, which incurs the risk of accumulation, or at extremes of weight or age [1].

Routine coagulation analysis includes activated partial thromboplastin time (aPTT), prothrombin time-international normalized ratio (PT-INR) and platelet count (Plc). aPTT is widely available but is generally considered suboptimal for monitoring agents other than unfractionated heparin (UFH) since it has low and varying sensitivity to anticoagulation caused by LMWH’s, direct thrombin (factor IIa, FIIa) and anti-factor Xa (anti-FXa) inhibitors such as dabigatran and rivaroxaban respectively [2]. In contrast to PT-INR, which is internationally standardized such that all PT-INR measurements on a single sample ought always to be the same, variation in aPTT exists between laboratories and reagents, complicating the use of this assay for guiding dosage of LMWH [3,4].

While anti-FXa activity assays are reliable determinants of the concentration of LMWH in the blood [5] and are established as a gold standard, they do not necessarily correlate well to the actual effect of the drug in vivo: they describe pharmacokinetics rather than pharmacodynamics. Tests of global coagulation such as aPTT and PT-INR are different from anti-FXa activity tests in that they reflect LMWH’s clinical effect [6,7].

Most of LMWH’s anticoagulative effect is provided by indirect inhibition of FXa which cleaves FII to FIIa, but like UFH they also show some indirect inhibition of FIIa itself. The anticoagulative effect of UFH and LMWH also depends on numerous other factors both pharmacokinetic and pharmacodynamic. Among these factors are that these drugs release tissue factor pathway inhibitor, and there are interindividual variations in heparin-binding proteins [8,9].

LMWH’s are produced through depolymerization of UFH resulting in shorter heparin fragments and a lower mean molecular weight. There are several brands of LMWH available, manufactured through different chemical methods. This results in various chemical structures in the saccharide fragments that are isolated [10]. LMWH’s with longer fragments, such as tinzaparin, tend to inhibit FIIa more strongly, while LMWH’s with shorter fragments, such as enoxaparin, exert more specific inhibition of FXa. The resultant pharmacodynamic differences are described by the FXa/FIIa ratio–see Table 1 [11,12].

**Table 1:** Molecular weight and anti-factor Xa and anti-factor IIa activities of heparin and commonly used low molecular weight heparins (LMWH).^a^

Agent	Trade name in Sweden	Mean molecular mass	Anti FXa/FIIa (anti-FXa IU/mg)	Anti FXa/FIIa ratio
Unfractionated heparin (UFH)	Heparin	15 kDa	193/193	1
Tinzaparin	Innohep	6.8 kDa	90/45	2.0
Dalteparin	Fragmin	6.0 kDa	130/52	2.5
Enoxaparin	Klexane	4.2 kDa	100/25	3.9
Fondaparinux	Arixtra	1.7 kDa	930/0	∞

^a^The shorter the LMWH fragments, the more specific the agent is for factor Xa [11].

*In vivo* studies have suggested that measurement of the area under the thrombin generation curve, the endogenous thrombin potential (ETP), could be superior to the aPTT in monitoring the effect of LMWH and UFH [9,13,14]. It measures the total and physiologically relevant amount of thrombin formed upon *in vitro* activation of coagulation, whereas global methods based on clot formation time measure are sensitive only when the amount of thrombin formed is small.

The aim of this study was to compare the correlation of anti-FXa activity and thrombin generation with aPTT using four different methods. This is relevant to our clinical practice where we are confronted with the simple question: which test should we order to ensure correct dosage of LMWH? The tests were run on both enoxaparin and tinzaparin, since they have different FXa/FIIa inhibition ratios. Our hypothesis was that increased concentrations of LMWH would give prolongations of the aPTT, increased anti-FXa activity and inhibit FIIa generation. Due to the higher FXa/FIIa inhibition ratio of enoxaparin we expected it to have a lesser effect on the aPTT and FIIa generation than tinzaparin at any given level of anti-FXa activity.

## Materials and Methods

Approval was obtained from the Regional Ethical Review Board (Lund, Protocol DNR 2010/482) and signed consent given by the volunteers.

### Blood sampling and preparation

Blood taken from 10 volunteers by venous puncture was collected in citrated tubes (BD Vacutainer, 4.5 ml, 0.109 M sodium citrate). The samples were diluted with enoxaparin or tinzaparin to concentrations of 0.0, 0.5, 1.0 and 1.5 anti-FXa international units (IU) per ml plasma by adding a volume of 30 μl LMWH (0, 10, 20 and 30 IU/ml in physiological saline) to each ml blood. This dilution assumed that plasma accounted for 60% of the blood volume. The samples were then incubated for 10 minutes at 37°C before those intended for plasma-based aPTT and thrombin generation tests were immediately centrifuged for 20 minutes (2000 rpm at −20°C). Plasma was separated and stored at −80°C for later analysis. Whole blood aPTT was measured using the free oscillation rheometry (FOR) and Hemochron Jr apparatuses within an hour of sampling.

The titrations of LMWH used were selected to cover therapeutic concentrations: the plasma level of LMWH for treatment of pulmonary embolism should be 0.5 to 1.1 IU/ml of anti-FXa activity for twice-daily dosing, or 0.8 and 1.6 IU/mL for once-daily dosing [15].

### FOR—aPTT

FOR was performed using a ReoRox 4 instrument (MediRox AB, Nyköping Sweden). The reference interval in healthy individuals is 26–35 seconds (s). Two hundred μl of whole blood was added to a cylindrical plastic cup and incubated with 200 μl of aPTT reagent (MRX931, MediRox AB) for 5 minutes. The reaction was started by recalcification with 200 μl CaCl 2.5 mmol/ml. The sample was set into free oscillation and the frequency and damping of the sample measured over time.

### Hemochron Jr–aPTT

Whole blood aPTT was analysed using the Hemochron Jr (International Technidyne Corporation, Edison, New Jersey) (HCJ) system with a reference interval of 26–36s. Seventy μl citrated blood was added to a disposable cuvette specifically for citrated blood. The instrument moves the blood back and forth within a test channel having mixed it with a reagent. Clotting is detected using a LED light source and optical detectors.

### Plasma-aPTT

Plasma-aPTT was performed using a BCS-XP analyzer (Siemens Healthcare Diagnostics, Marburg, Germany) with two reagents, Actin FSL (Siemens) with a reference interval of 26–33s and PTT- Automat (Stago, Asnières, France) with a reference interval of 28–45s. For clinical reasons, aPTT’s of more than 150s are recorded as ‘>150s’. Coagulation is initiated with a contact activator and phospholipids in the reagent and after recalcification with CaCl_2_, the coagulation time is measured in seconds through optical detection.

### Anti-FXa activity

Anti-FXa activity was analysed on a BCS-XP with Coamatic Heparin (Chromogenix, Instrumentation Laboratories, Bedford, USA). This method is based on the inhibition of FXa by its forming inactive complexes with the antithrombin. LMWH accelerates this process such that the concentration of LMWH can be determined by the addition of a substrate which releases a colour upon cleavage by the remaining FXa, which has not bound to antithrombin [16].

### Thrombin generation

Thrombin generation was measured over time on a Ceveron Alpha (Technoclone, Vienna, Austria). The concentration of thrombin is measured with a fluorescent peptide substrate, which is cleaved by thrombin to release a fluorophore. Coagulation is initiated through the addition of tissue factor and phospholipids. The rate of thrombin generation is measured over time resulting in a thrombin formation curve. The area under the curve is calculated to give the ‘endogenous thrombin potential’ (ETP), which represents the total amount of thrombin generated.

We initially used the TGA RB (also manufactured by Technoclone) reagent, which contains 2pM human recombinant tissue factor. Due to the results from these analyses (see results section) we repeated the measurements using the TGA RC reagent, which contains a higher concentration of tissue factor (5pM).

### Statistical analysis

All statistical analysis was performed using the R software (version 3.0.3, www.r-project.org). The Wilcoxon signed rank test was conducted on each pair of contiguous groups of LMWH concentrations to test whether there was a significant difference in aPTT between the two groups. It was chosen over the standard Student’s t-test due to the relatively small number of samples in this study, where the Wilcoxon signed rank test has proven to be more powerful [13]. P<0.05 was considered statistically significant. Box-plots were drawn where the box represents the interquartile range and the whiskers represent the range excluding outliers, defined as being more than 1.5 times the interquartile range from the upper or low quartiles. Spearman’s ranked correlation test was performed to quantify the correlation between each aPTT method and the anti-FXa activity. It was chosen instead of Pearson’s product-moment correlation due to the samples of the full group not being normally distributed, which is a requirement for using the Pearson’s product moment correlation.

Since the plasma-based methods (ActinFSL and PTT-Automat) gave numerical results for aPTT’s of 150s or less, we registered results of ‘>150s’ as 150s in our data set. Due to 8 of the 10 subjects having at least one plasma-aPTT of ‘>150s’ in the presence of 1.5 IU/ml of tinzaparin, we excluded all data from this concentration of LMWH when calculating correlation factors.

## Results

Ten volunteers were included, all healthy men with ages ranging from 23–62 years. None had a known coagulation disorder. Our results are available as raw data in S1 File.

aPTT exhibits a linear dose-response to LMWH; there is variation between assays.

**Figure 1:**
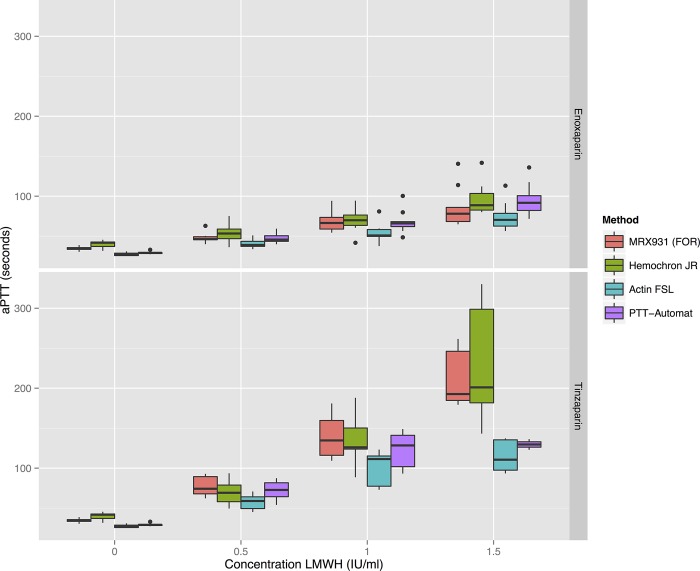
Boxplots of the four aPTT methods’ results for enoxaparin and tinzaparin in three different concentrations. When comparing the contiguous groups with the same aPTT-method but different concentrations of LMWH, a significant difference in aPTT was found in all cases. Statistical significances between classes are shown in S1 File.

Fig. 1 shows that within the range tested, there is a linear dose-response relationship for each LMWH measured with the four different aPTT tests. With every increase of 0.5 IU/ml in LMWH concentration there was a statistically significant increase in aPTT for all the tests.

There were some significant differences between different methods’ aPTT results. The Hemochron Jr showed a tendency to give longer aPTT’s but the only reagent which consistently gave different results compared to the other methods was the plasma-based ActinFSL, which gave the shortest mean aPTT for all concentrations of LMWH. Which significant differences were present between reagents at different concentrations is shown in S2 File.

### Tinzaparin prolongs aPTT more than enoxaparin

**Figure 2:**
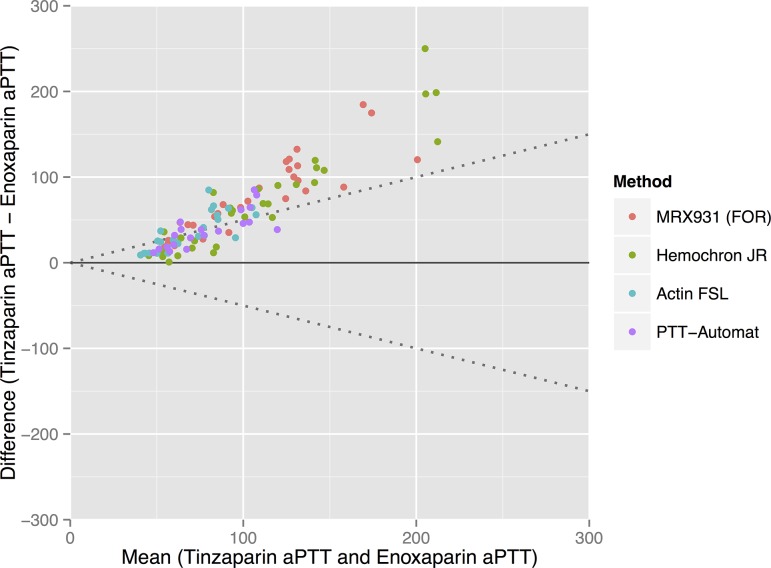
A comparison of enoxaparin and tinzaparin’s relative effects on aPTT. Bland-Altman plot showing that the aPTT’s induced by tinzaparin ranged from on average 49% more than enoxaparin (when measured using the PTT-Automat reagent) to 66% more than enoxaparin (when measured using the MRX931 reagent (FOR: free oscillation rheometry).

Tinzaparin gave significantly longer aPTT’s than enoxaparin at any given dosage of anti-FXa activity—see Figs. 1 and 2. Complete tables of this data in S3 File show that the mean aPTT at a concentration of 1 IU/mL of LMWH varied from between 54s (SD 11.4) and 69s (SD 13.8) for the enoxaparin samples to between 101s (SD 21.0) and 140s (SD 27.6) for the tinzaparin samples. The standard deviation of the aPTT results increases with increasing concentrations of LMWH.

### APTT is linearly correlated to anti-FXa activity, which is strongly correlated to the concentration of LMWH

**Figure 3:**
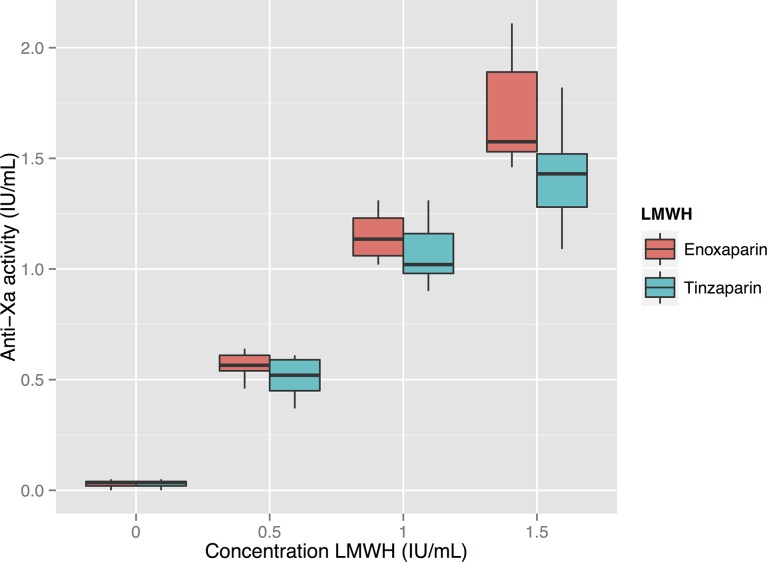
Relationship between measured anti-FXa and the concentration of low molecular weight heparin (LMWH). There is a strong linear correlation between anti-FXa results and the concentration of LMWH (dosed in anti-FXa units per ml). For enoxaparin and tinzaparin the correlation coefficients R_s_ are 0.97 and 0.96 respectively.

**Figure 4:**
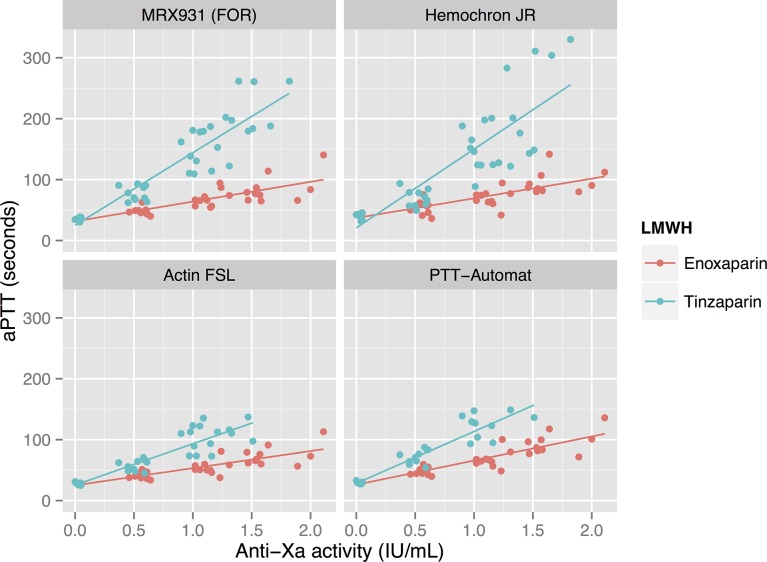
Correlations between aPTT and anti-FXa at varying concentrations of enoxaparin and tinzaparin, using various reagents. Anti-FXa activity reflects the concentration of LMWH and the aPTT correlated well to this measure. The correlation for Hemochron Jr is slightly weaker than for the other reagents (see Table 2).

Anti-FXa activity showed a strong dose-response to both enoxaparin and tinzaparin—see Fig. 3. There is a linear relationship between the anti-FXa activity and aPTT with good correlations: see Fig. 4 and Table 2. Hemochron Jr had the lowest correlation coefficients (RS 0.67 and 0.85 for enoxaparin and tinzaparin respectively) while the other methods were correlated more strongly (RS between 0.80 and 0.91). ActinFSL’s regression line was flatter than the other methods, reflecting the fact that ActinFSL gives lower values of aPTT compared to the other methods.

**Table 2:** Explanatory to Fig. 4: Correlation and regression between aPTT and Anti-FXa for enoxaparin and tinzaparin.

	Enoxaparin		Tinzaparin	
Reagent	R_S_	Regression line	R_S_	Regression line
MRX931 (FOR)	0.88	y = 32x + 32	0.88	y = 119x + 25
Hematochron Jr	0.67	y = 32x + 37	0.85	y = 129x + 21
ActinFSL	0.80	y = 28x + 25	0.89	y = 68x + 25
PTT-Automat	0.86	y = 39x + 26	0.91	y = 91x + 27

FOR: free-oscillation rheometry.

### Thrombin generation shows a negative exponential dose-response to increasing doses of LMWH

Thrombin generation measured using the TGA RB reagent was strongly inhibited by all concentrations of LMWH and showed an unacceptable degree of variation between results at each concentration—see diagram in S3 File.

**Figure 5:**
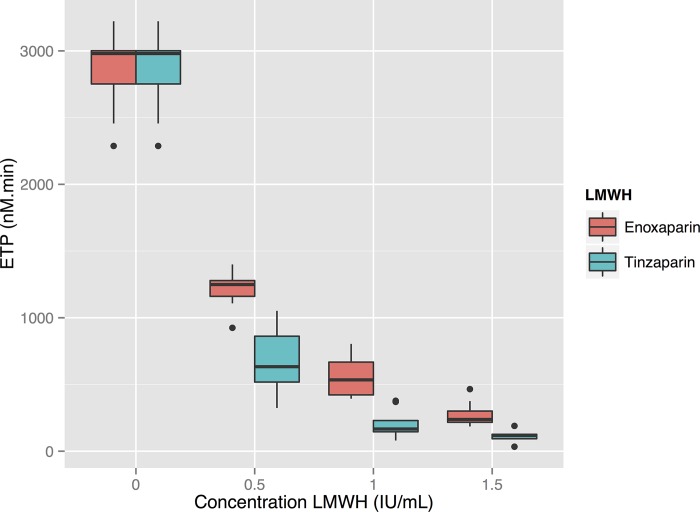
Thrombin generation at increasing concentrations of LMWH^a^, measured using the TGA RC reagent. Thrombin generation is inhibited in a negative exponential manner by increasing doses of LMWH. There is a significant difference between Endogenous Thrombin Potential (ETP) at all contiguous concentrations and between ETP for the two reagents at each individual concentration (P<<0.05). ^a^Low Molecular Weight Heparin. ^b^ETP: Endogenous Thrombin Potential.

Enough plasma remained to run the tests again using the TGA RC reagent on eight of the ten subjects’ plasma. With this reagent the ETP showed a negative exponential relationship to the dose of LMWH, with tinzaparin exhibiting stronger inhibition of thrombin generation than enoxaparin: the mean ETP at a LMWH concentration of 1.0 anti-FXa IU/ml was 560 nM.min (SD 152) for enoxaparin and 206 nM.min (SD 104) for tinzaparin (see Fig. 5). Differences between the different concentrations as well as the two LMWH’s were all statistically significant.

### APTT shows a negative linear correlation to thrombin generation’s logarithm

**Figure 6:**
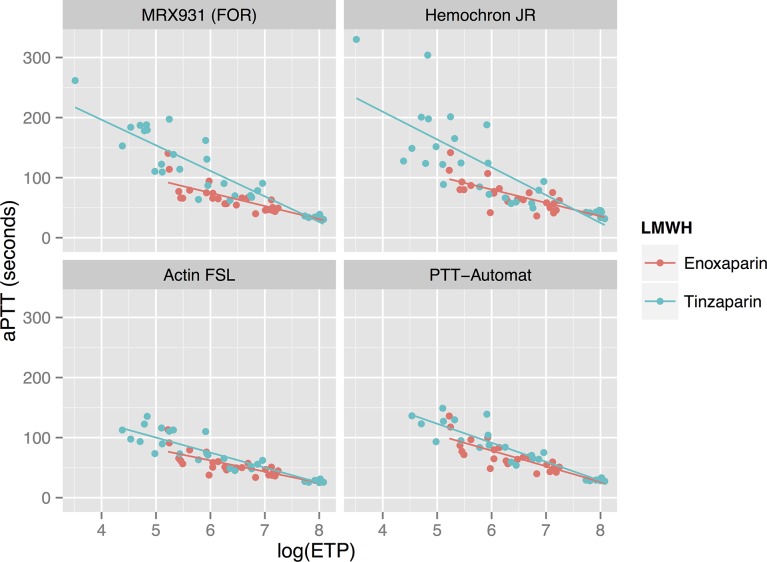
Relationship between aPTT measured using various reagents, and the logarithm of thrombin generation. See Table 3 for correlation factors and regression lines. There is a negative linear relationship between aPTT and log_10_(ETP). Tinzaparin results in prolonged aPTT results compared to enoxaparin in the whole blood analyses (FOR and Hemochron Jr) at any given level of thrombin generation.

See Fig. 6 and Table 3. The logarithm of ETP measured using the TGA RC reagent showed a strong linear negative correlation to the aPTT, as measured using all apparatuses other than Hemochron Jr. For enoxaparin the correlation coefficient RS was between −0.80 and −0.88 while for tinzaparin RS was between −0.87 and −0.92. Due to HCJ’s aPTT results being more variable at higher concentrations of LMWH, the correlation between HCJ-aPTT and log_10_(ETP) was weaker (Rs −0.62–0.85). In the whole blood assays (FOR and HCJ) there was a tendency for tinzaparin to elevate the aPTT more than enoxaparin at any given level of inhibition of thrombin generation.

aPTT: activated partial thromboplastin time. ETP: endogenous thrombin potential. FOR: Free-oscillation rheometry.

**Table 3:** Explanatory to Fig. 6.

	Enoxaparin		Tinzaparin	
Reagent	R_S_	Regression line	R_S_	Regression line
MRX931 (FOR)	-0.88	y = −0.017x + 80	-0.87	y = −0.042x + 147
Hematochron Jr	-0.62	y = −0.017x + 85	-0.87	y = −0.043x + 153
ActinFSL	-0.83	y = −0.015x + 66	-0.92	y = −0.027x + 100
PTT-Automat	-0.80	y = −0.021x + 85	-0.92	y = −0.035x + 125

FOR: free-oscillation rheometry.

### Anti-FXa activity shows a negative linear correlation to thrombin generation’s logarithm

**Figure 7:**
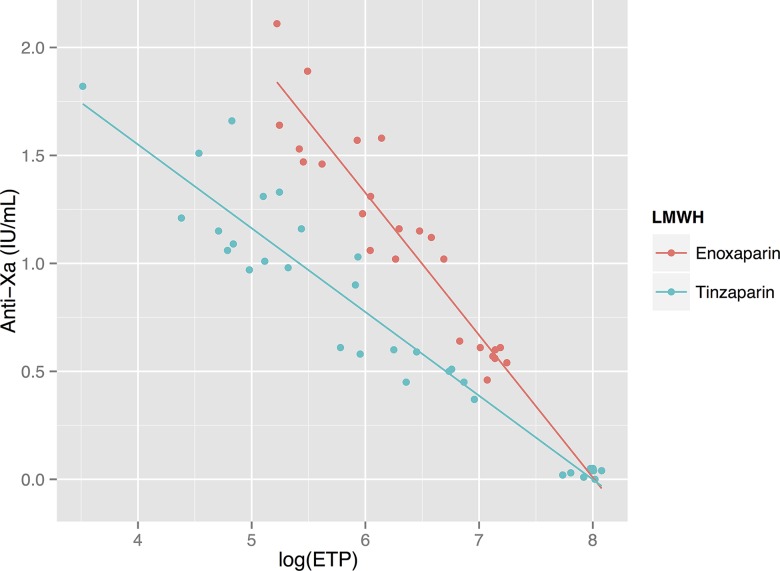
Relationship between anti-FXa activity and thrombin generation. Anti-FXa activity is strongly negatively correlated to the logarithm of ETP (Endogenous Thrombin Potential): R_s_ for enoxaparin and tinzaparin are −0.93 and −0.94 respectively. Anti-FXa activity is more prolonged by enoxaparin than tinzaparin at any given level of thrombin generation.

Correlations between the log_10_(ETP) and anti-FXa activity were also very strong for both LMWH’s (RS −0.93 and −0.94 for enoxaparin and tinzaparin respectively)—see Fig. 7. In contrast to aPTT, which was elevated more by tinzaparin in the whole-blood samples, enoxaparin resulted in a somewhat more elevated anti-FXa for any given level of inhibition of thrombin generation, which is to be expected since it has an anti-FXa/FIIa ratio of 3.9 compared to tinzaparin’s ratio of 2.0.

## Discussion

### Enoxaparin and tinzaparin vary in their pharmacological properties and the anticoagulative effect of LMWH may vary between patients

Our results support previous work showing that the anti-FXa assay is an excellent test for determining the concentration of LMWH in plasma [5]. It does not, however, reflect the absolute anticoagulative effect of the drugs it measures despite being strongly correlated to aPTT in this study (excluding Hemochron Jr: R_S_ = 0.80–0.91). One recent study conducted on 149 patients receiving unfractionated heparin for various indications found the correlation between the anti-FXa activity and aPTT to be much weaker (r = 0.61), which was attributed to variation in FII and FVIII [17]. We suggest that although the anti-FXa test should be used to dose LMWH’s in patients with unpredictable pharmacokinetics: children, the obese, those in renal failure; more functional tests of anticoagulation are required to monitor the actual effect of these drugs [18,19].

Since both enoxaparin and tinzaparin were dosed in anti-FXa units in our study, the anti-FXa effect of each drug at each concentration was the same—see Fig. 3. Tinzaparin attenuated the ETP more potently, and prolonged aPTT more than enoxaparin at equivalent levels of anti-FXa activity—see Figs. 1 and 5. This has been observed in previous studies and would appear to be due to tinzaparin’s stronger inhibition of FIIa [20]. We would like to emphasize that it is illogical and confusing that despite tinzaparin’s greater anti-FIIa effect, it is clinically administered in anti-FXa IU/mL/kg while enoxaparin is administered in mg/kg.

### aPTT is prolonged by LMWH; there is variation between methods and reagents

Due to there being no standardisation of reagents used for aPTT, each laboratory has its own reference interval for their own specific reagent, instruments and population. This leads to clinical problems interpreting aPTT results in the clinical context, confounded by the fact that prolonged aPTT can be caused by factors other than coagulopathy *per se.* The time between sampling and analysing and the fasting state of the patient may influence the aPTT, as can a high haematocrit, which results in a lower plasma volume per ml blood taken at venepuncture such that the ratio of citrate to plasma is increased, prolonging the aPTT [21]. Our results demonstrate that there is significant variation between the different methods even when time to sampling and hydration state of each individual subjects was the same for all the tests.

The aPTT’s produced using the ActinFSL reagent were lower than the values provided by the other methods (see Fig. 4). This does not mean that ActinFSL is a less useful assay than the other tests since it correlates just as strongly to the concentration of LMWH, aPTT and anti-FXa activity. Clinicians must, however, be aware that changing to ActinFSL from one of the other reagents will result in a shorter aPTT despite an unchanged level of anticoagulation.

### Patient-near monitoring with aPTT

The two whole blood methods (FOR and Hemochron Jr: HCJ) can be used in the point-of-care setting. The Hemochron Jr apparatus in particular is portable, robust and easy to use. At lower concentrations of LMWH it has a similar profile to the FOR in terms of sensitivity and variance of results—see Fig. 1—and may therefore be suitable for monitoring thrombosis prophylaxis in patients at risk of accumulation of LMWH, for example in renal failure. The Hemochron Jr did, however, have a lower correlation coefficient to anti-FXa activity and ETP than the other aPTT methods in our study, and a greater variance than the FOR apparatus at higher concentrations—see Figs. 1 and 4. The more cumbersome FOR apparatus would therefore be more appropriate for monitoring higher concentrations of LMWH where the dose must be titrated to balance the risk of thrombosis and haemorrhage. It would be of clear clinical interest to compare FOR with the activated clotting time (ACT), which has shown some *in vitro* potential for monitoring LMWH [22].

### Thrombin generation measures both anti-FXa and anti-FIIa activity and therefore has clinical potential

Generation of thrombin (FIIa) is a key event in the coagulation process, and is dependent upon FXa. Measurement of thrombin generation therefore reflects both LMWH’s main effects: indirect inhibition of FXa and direct inhibition of thrombin itself.

The negative exponential relationship that we demonstrated between increasing concentration of LMWH and thrombin generation, and thrombin generation’s very strong correlation to anti-FXa activity mean that it would also be useful for monitoring the effect of LMWH. When al Dieri et al. found that the ETP had a much higher sensitivity (43% for aPTT and 93% for ETP) for low doses of UFH, they attributed this to the exponential dose-response curve of the ETP resulting in a high reduction of the ETP already at very low doses of UFH, rather than a higher accuracy or more stable normal value than the aPTT [13].

Although thrombin generation appears to be a suitable method to estimate the anticoagulative effect of LMWH, it is noteworthy that the aPTT’s produced by tinzaparin and enoxaparin at equivalent levels of thrombin generation differ (see Fig. 6). This supports the concept that the two LMWH’s affect global coagulation by mechanisms other than pure thrombin-inhibition.

### Strengths and weaknesses of the study

We are not aware of any previous study where several methods of aPTT measurement, anti-FXa activity and thrombin generation have simultaneously been tested with low molecular weight heparins with differing anti-FXa/anti-FIIa ratios. It is of particular clinical interest to bring to attention the finding that the anti-FXa is not a functional measure of anticoagulation.

Among the weaknesses of this study is that it is an *in vitro* study—enoxaparin and tinzaparin were administered directly into citrated blood samples rather than subcutanously into our volunteers. Another weakness is that all samples were taken from healthy men and not patients.

It is known that there is an interindividual variation in response to intravenous administration of unfractionated heparin to patients and after subcutaneous administration of LMWH to healthy volunteers [8,9]. We cannot in this study be sure whether the greatly increased standard deviation of aPTT results at higher levels of LMWH was due to the methods used to measure aPTT or to variation in the subjects’ responses. We can, however, be sure that there was a significant difference between aPTT methods.

## Conclusion

aPTT and anti-FXa display linear dose-responses to LMWH, although aPTT is less strongly correlated to the dose of LMWH than anti-FXa activity. There is some variation between aPTT assays. Tinzaparin increases aPTT and decreases thrombin generation more than enoxaparin at any given level of anti-FXa activity, which should lead to caution in interpreting clinical anti-FXa results. Thrombin generation with tissue factor-rich activator is a promising method for monitoring LMWH’s.

## Supporting Information

S1 File Raw data as tabulated text file. (TXT)

S2 File Modification of Fig. 1 displaying which aPTT methods gave statistically different results at each concentration of low molecular weight heparin (LMWH). Horizontal bars indicate a significant difference in aPTT results given by reagents at each concentration of LMWH as tested by the Wilcoxon signed rank test (P<0.05). The aPTT results given by the ActinFSL reagent were significantly different from the other reagents at almost all concentrations.
(TIFF)

S3 File Thrombin generation at increasing concentrations of LMWH^a^, measured using the TGA RB reagent. ETP (Endogenous Thrombin Potential) is strongly inhibited by LMWH. ^a^Low Molecular Weight Heparin.
(TIFF)
