# Supplementary figures and images for: Monitoring Low Molecular Weight Heparins at Therapeutic Levels: Dose-Responses of, and Correlations and Differences between aPTT, Anti-Factor Xa and Thrombin Generation Assays
Source: PLoS One. 2015 Jan 27;10(1):e0116835. doi: 10.1371/journal.pone.0116835 (PMC4308107; doi:10.1371/journal.pone.0116835)

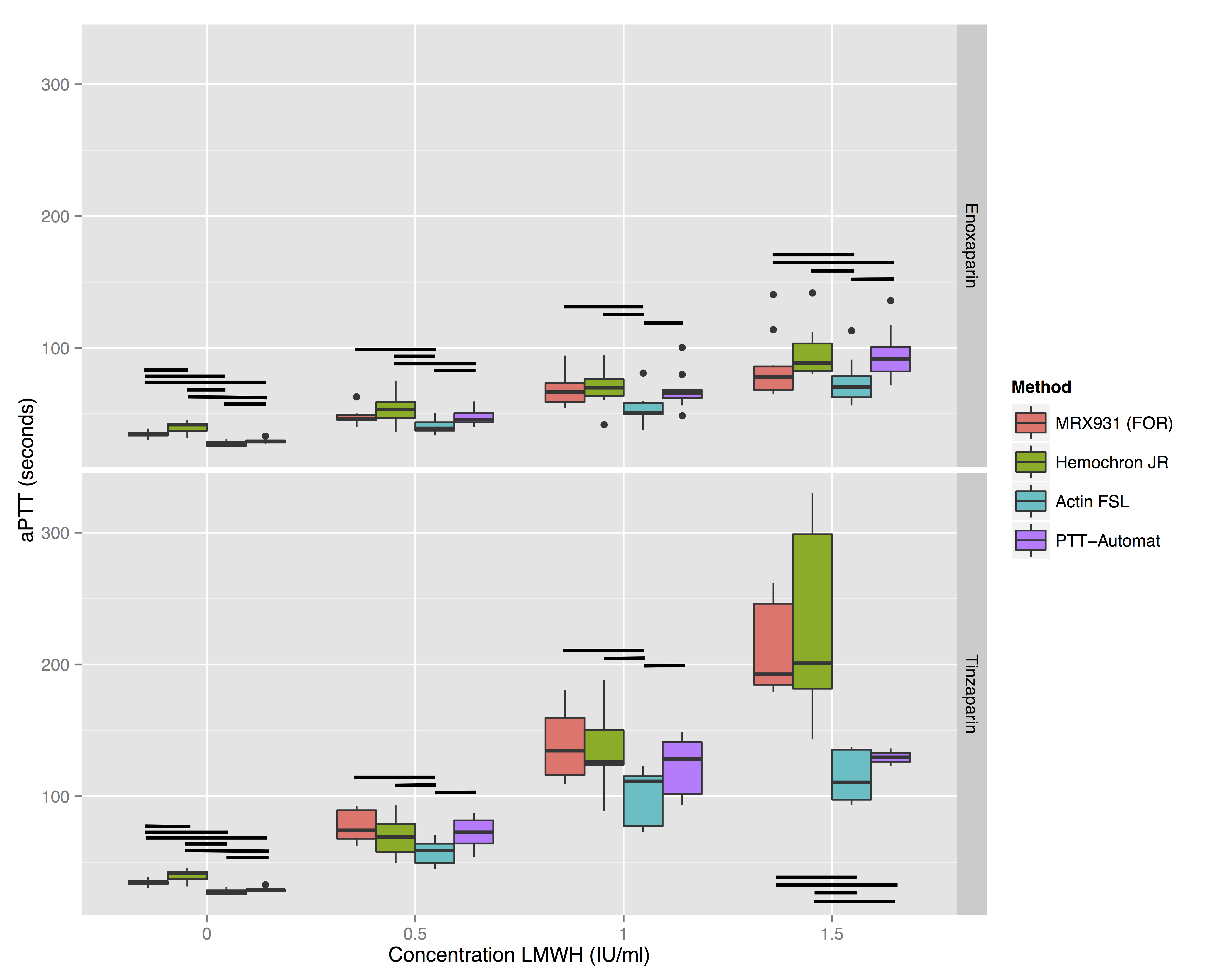

Supplement: S2 File — Horizontal bars indicate a significant difference in aPTT results given by reagents at each concentration of LMWH as tested by the Wilcoxon signed rank test (P<0.05). The aPTT results given by the ActinFSL reagent were significantly different from the other reagents at almost all concentrations. (TIFF) [file pone.0116835.s002.tiff]

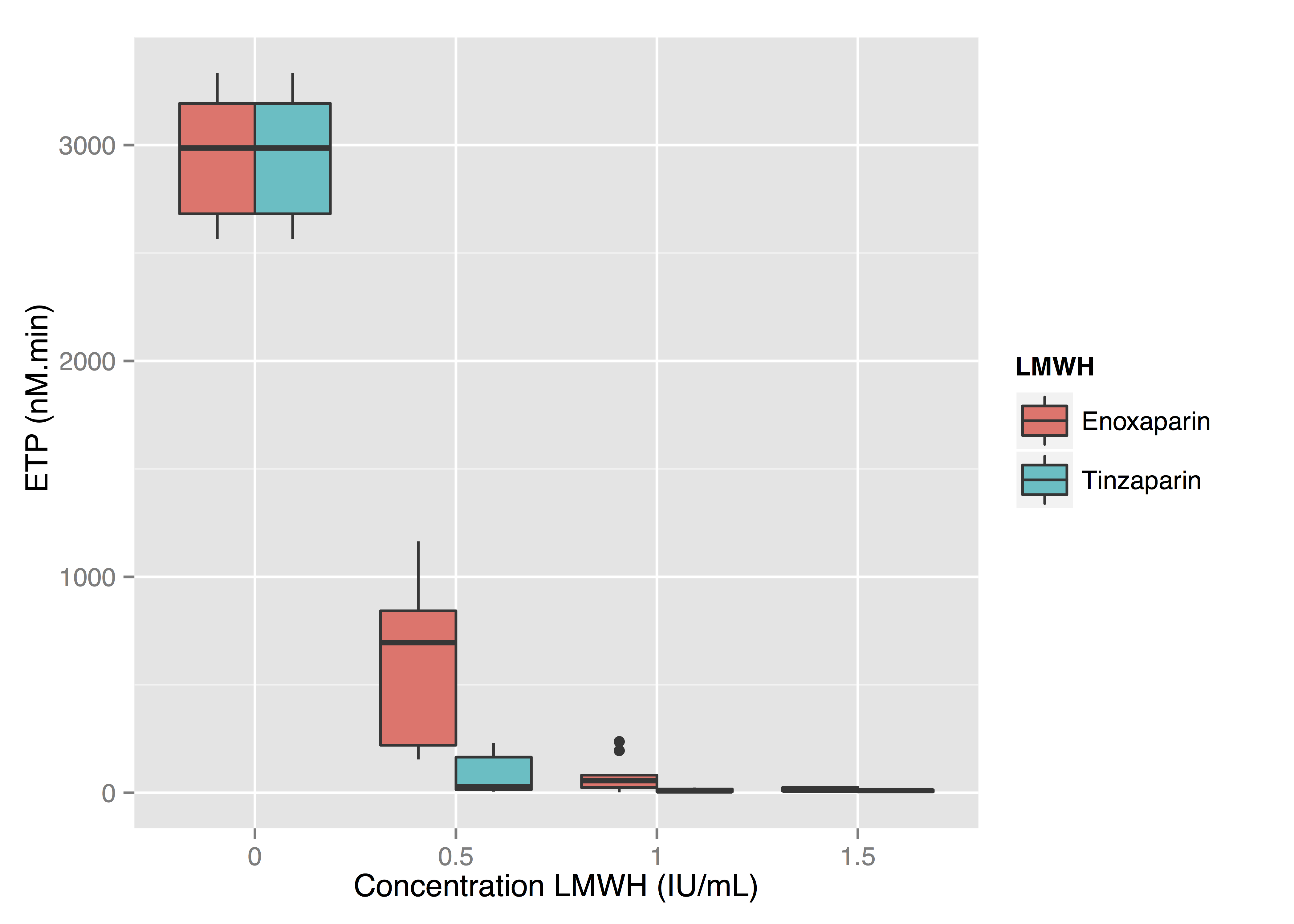

Supplement: S3 File — ETP (Endogenous Thrombin Potential) is strongly inhibited by LMWH. aLow Molecular Weight Heparin. (TIFF) [file pone.0116835.s003.tiff]
